# Targeting the inflammation in HCV-associated hepatocellular carcinoma: a role in the prevention and treatment

**DOI:** 10.1186/1479-5876-8-109

**Published:** 2010-11-03

**Authors:** Giuseppe Castello, Susan Costantini, Stefania Scala

**Affiliations:** 1 Oncology Research Centre of Mercogliano (CROM), Mercogliano (AV), Italy; 2 National Cancer Institute of Naples, "G. Pascale Foundation", Naples, Italy

## Abstract

Epidemiological, preclinical and clinical studies demonstrated that chronic inflammation induced by hepatitis C virus (HCV) is crucial in hepatocellular carcinogenesis. The interaction between hepatocytes and microenvironment regards virus, inflammatory and immunocompetent cells, chemo- and cyto-kines, reactive oxygen species (ROS) and nitric oxide (NO), generating cell transformation. We suggest hepatocarcinoma (HCC) as a model in which the targeting of microenvironment determine neoplastic transformation. The present review focuses on: the role of inflammation in carcinogenesis, the clinical impact of HCC and the inadequacy of the actual therapy, the chemoprevention targeting the microenvironment.

## HCC epidemiology

Hepatocellular carcinoma (HCC) accounts for > 5% of all human cancers and for 80% - 90% of primary liver cancer. It is a major health problem worldwide being the fifth most common malignancy in men and the eighth in women; the third most common cause of cancer-related death in the world. Moreover early diagnosis is uncommom and medical treatments are inadeguate [1].

Yearly 550,000 people worldwide die for HCC, with a 2:1 ratio for men versus women. Its incidence is increasing dramatically, with marked variations among geographic areas [2], racial and ethnic groups, environmental risk factors [3,4]. The estimated annual number of HCC cases exceeds 700,000, with a mean annual incidence of 3-4% [2]. Most HCC cases (> 80%) occur in either sub-Saharan Africa or in Eastern Asia (China alone accounts for more than 50% of the world's cases) [2]. In the United States (US) HCC incidence is lower than other countries (0.3/100,000) even if there has been a significant and alarming increase in the incidence of HCC in the US, from 1.3 in the late 70s' to 3 in the late 90s', due to HCV infection. In 2008, 21,370 new cases of HCC and intrahepatic bile duct cancer were estimated with 18,410 deaths [2]. In Europe, Oceania and America, chronic hepatitis C and alcoholic cirrhosis are the main risk factors for HCC. The main risk factor for HCC development in patients with hepatitis C is the presence of cirrhosis. Among patients with hepatitis C and cirrhosis, the annual incidence rate of HCC ranges between 1-8%, being higher in Japan (4-8%) intermediate in Italy (2-4%) and lower in USA (1.4%) [5]. Analysis of mortality from HCC in Europe confirmed large variability, with high rates in France (6.79/100,000) and Italy (6.72/100,000) due to hepatitis C virus (HCV) during the 1960 s and 1970 s [6]. Southern Italy has the highest rates of HCC in Europe [7].

## HCC etiopatogenesis

HCC is unique among cancers occurring mostly in patients with a known risk factor: ninety percent of HCCs develop in the context of chronic liver inflammation and cirrhosis [1]. Hepatitis B (HBV) and C (HCV) viruses are the major cause of liver disease worldwide. Fortunately, the hepatitis B virus vaccine has resulted in a substantial decline in the number of new cases of acute hepatitis B among children, adolescents, and adults in western countries since the mid-1980 s. This success is not duplicable for HCV where active or passive vaccination is not available yet. Therefore, the present and next future HCC history will be mainly related to HCV infection. The incidence of HCV infection is hard to quantify since it is often asymptomatic. The World Health Organization estimates that 3% of the world's population - more than 170 million people - are chronically infected (3-4 million new infections every year). Therefore, a tremendous number of people are currently at elevated risk for HCC and its early diagnosis (when surgical intervention is possible) may significantly affect the patients prognosis [8].

However it is possible also a direct carcinogenesis by hepatitis viruses, without a cirrhotic step [5,9]. In particular, it was reported that patients without cirrhosis were younger, survived longer than patients with cirrhosis (P < 0.0001) and had a better 5-year survival experience [10]. The action of some viral proteins (mainly the HCV core protein and the HBV X protein) [11] or insertional mutagenesis in the case of HBV [12,13] were suggested as potential mechanisms to induce HCC.

In contrast to HBV, HCV does not integrate into the host genome and does not contain a reverse transcriptase. In particular, in the infected subjects both viruses trigger an immune-mediated inflammatory response (hepatitis) that either clears the infection or slowly destroys the liver [14].

**Figure 1:**
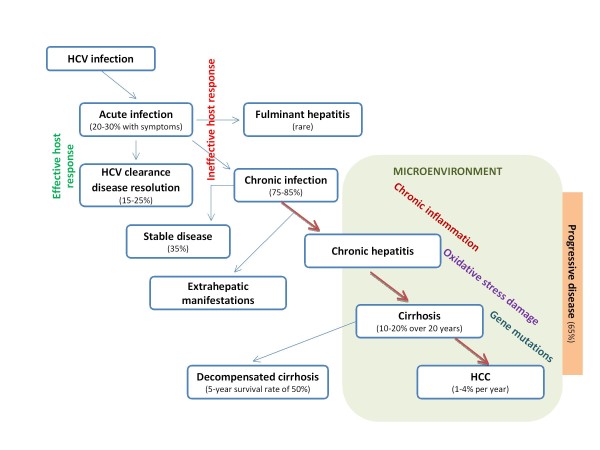
**Evolution from HCV infection to HCC**.

Effective HCV immunity is limited by the high variability of virion genome; HCV virions turn over rapidly (with a half-life of about 3 h), and up to about 10^12 ^complete viruses are produced per day in an infected person [15]. About 80% of newly infected patients develop chronic infection; an estimated 10% to 20% will develop cirrhosis and 1-5% proceeds to end-stage liver cancer over a period of 20 to 30 years (Figure 1). In the case of HCV, HCC is invariably observed as a complication of cirrhosis, whereas in the case of HBV HCC is often found in non-cirrhotic liver. Therefore, the hepatic fibrosis dramatically increases the incidence of HCC [16].

## Anti-HCV immune response

### Innate response

In the blood of infected patients, HCV is associated with blood lipoprotein VLDL, LDL, and HDL; although the virus binds to different molecules it requires tetraspanin CD81, the scavenger receptor class B type I (SR-BI), the tight junction proteins claudin (CLDN1) and occludin [17-20] to entry into hepatocytes. The host response is triggered when a pathogen-associated molecular pattern (PAMP), presented by the infecting virus, is recognized and engaged by specific pathogen recognized receptor (PRR), as the Toll-like receptors (TLRs) [20,21]. Early after infection, the immune system reacts to viral RNA through a signaling cascade which results in interferon (IFN) production [22].

Two main pathways lead to an IFN response. One is mediated by retinoic acid inducible gene-I (RIG-I) retinoic acid/MDA5 while MyD88 (myeloid differentiation primary response gene 88) activates the other. RIG-1 senses triphosphorylated single stranded HCV RNA and MDA5 recognizes dsRNA. Both act on Interferon promoter stimulator 1(IPS-1) that transmits the activation signal to IKKe and TANK-binding kinase-1 (TBK-1). These two kinases in turn phosphorylate the interferon regulator factor-3 (IRF-3) that activates the IFN-β promoter [23].

Double-stranded HCV RNA is also recognized by TLR-3, which activates IKKe/TBK-1, via TRIF (TIR-domain-containing adapter-inducing interferon-β) joining the RIG-I/MDA5 pathway. In the other pathway, TLR7 senses single-strand HCV RNA and via the MyD88 adaptor protein activates IRAK4/IRAK1. These kinases stimulate IFN-χ synthesis via the transcription factor of interferon response factor 7. MyD88 is a universal adaptor protein being used by other TLRs (except TLR-3) to activate the transcription factor NF-kB. This leads to the expression of IFN-α/β, other cytokines/chemokines and facilitates leucocyte recruitment. Secreted IFN-α/β bind to IFN receptors to stimulate the Jak-STAT pathway, resulting in the induction of over 300 genes. Several IFN-induced proteins (the protein kinase R, the RNAspecific adenosine deaminase 1, the 2'-5' oligoadenylate synthetases (2-5 OAS)/RNaseL system53 and P56) were reported to have anti-HCV activities.

#### HCV strategies to evade IFN mediated response

HCV evades INF-mediated antiviral activity using several different strategies [23]. A classic example of a PAMP is double stranded RNA and the best-described PRRs in hepatocytes are RIG-1 and TLR3, a toll-like receptor. When these PRRs detect viral invaders, such as HCV, they trigger signaling cascades that result in the transcription of IFNs and key messenger cytokines that activate host defenses. RIG-1 is activated by the binding of viral RNA, which enables RIG-1 to bind to IFN promoter stimulator 1(IPS-1) and trigger a signaling cascade that results in IFN transcription. IPS-1 is normally localized to the membranes of mitochondria but the HCV NS3-4a protease cleaves IPS-1, which causes it to delocalize from the mitochondrial membrane and prevents RIG-1 signaling. Importantly, liver-tissue samples from patients infected with HCV demonstrate IPS-1 delocalization, which suggests that this mechanism is clinically relevant. NS3-4a has also been demonstrated to inactivate the cellular protein toll-interleukin-1 receptor domain-containing adaptor inducing IFN (TRIF). TRIF is an adaptor protein that is a critical component of the TLR3 signaling pathway. By cleaving IPS-1 and inactivating TRIF, HCV disrupts the ability of a cell to detect its presence, as a consequence, IFN production is diminished and host defenses are impaired [23].

HCV is also able to interfere with specific host defenses that are induced by IFNs. The cellular factor PKR shuts down the production of proteins in infected cells. This strategy is a cellular mechanism that prevents cells from being used as factories for virus production. The ability of NS5a to inhibit PKR seems to be HCV-genotype specific and could be one reason for the greater sustained viral response (SVR) rate observed in patients infected with genotype 2 than in those with other HCV genotypes [24].

#### Natural Killer cells

HCV again employs multiple mechanisms to escape the NK cell response. Dysfunctional NK cells were found both in the periphery and in the liver during HCV infection. First, HCV E2 binding to CD81 directly inhibited NK cell activity. Second, HCV core protein stabilized the HLA-E expression and inhibited cytolysis of NK cells. Third, the transforming growth factor β (TGF-β) upregulates the inhibitory dimer of CD94/NKG2A on NK cells in HCV-infected patients. In addition, dendritic cells (DC) sense virus infection via toll-like receptors (TLR) or retinoic acid inducible gene-I (RIG-I), resulting in the secretion of type-I interferons (IFN) and inflammatory cytokines. In Myeloid DC from HCV-infected patients the levels of TLR/RIG-I-mediated IFN-β or TNF-α induction are lower than those in uninfected donors. These results suggest that the signal transduction in the downstream of TLR/RIG-I in MDC is profoundly impaired in HCV infection. In response to IFN-α, DC are able to express MHC class-I related chain A/B (MICA/B) and activate natural killer (NK) cells following ligation of NKG2 D. Interestingly, DC from HCV-infected patients are unresponsive to exogenous IFN-α to enhance MICA/B expression and fail to activate NK cells [25].

Furthermore, modulation of TLR-mediated signaling in a macrophage cell line expressing HCV proteins was identified. Clinical trials showed that agonists of TLR3, TLR4, TLR7, TLR8, and TLR9 were potent inducers of antiviral activity. These data indicate that stimulation of certain TLRs may have benefit on restoration of innate and adaptive immunity in chronic HCV infection. Therefore, cross talks between DC, NK, and NKT cells are critical in shaping subsequent adaptive immune response against HCV.

#### Plasmacytoid dendritic cells (PDCs)

Interestingly, patients who are chronically infected with HCV have decreased numbers of PDCs compared with healthy controls. Furthermore, PDCs from HCV-infected patients produce less IFN when stimulated compared with PDCs from healthy individuals [23]. In HCV-infected liver the plasmacytoid dendritic are responsible for the production of interferon I (IFN-I) binding to the IFN-α/β receptor activates the JAK/STAT pathway, which results in the induction of IFN-stimulated genes (ISGs) [26].

Host factors are involved in innate immune response. Certain human leukocyte antigen (HLA) allelic variants of DRB1 and DQB1 are associated with spontaneous HCV clearance, being polymorphisms in the interleukin (IL)-12B gene. Three landmark genome-wide association studies (GWAS) recently identified IL-28B gene locus is pivotal to the pathogenesis of HCV infection. Polymorphisms near the IL-28B gene not only predicted treatment-induced and spontaneous recovery from HCV infection, but they also explained, to some extent, the difference in response rates between Caucasians and African Americans to standard therapy with pegylated interferon and ribavirin [27].

### Specific immunity

Immature dendritic cells (iDCs) present in the liver express low levels of MHC class II and co-stimulatory molecules (CD80 and CD86), lacking CD1a, producing suppressive cytokines such as interleukin 10 (IL-10) [28]. Mature DCs (mDC) release a variety of cytokines (IL-12, TNF-α, IL-18, or IFN-α) that act on NK cells, mDCs prime T_H_0 cells and induce inflammatory CD4+ T-helper type 1 (T_H_1) cells and CD8+ CTL responses. Antigen-specific T_H_1 cells produce IL-2 and IFN-γ. IL-2-activated NK cells kill iDCs, thus limiting (down-regulating) the immune response. Impairment of DCs in NK cell activation may be responsible for the failure of an adequate immune response against HCV in the early phase of primary HCV infection [29,30] through secretion of suppressive cytokines IL-10 and TGF-β1 [31-33] as well as insufficient production of IFN-γ by NK cells in response to IL-12 and IL-15 activation [34]. A significant proportion of hepatic T cells are either CD4+ or double negative (CD4-CD8-) and express receptors typical of both NK cells (CD16+, CD56+, CD161+) and T-cells (T-cells receptors, TCRs). These cells, called NKT, constitute a conserved T-cell sublineage with unique properties; NKT cells express a limited αβTCR repertoire (i.e. an invariant V24-J15 TCR) and recognize glycolipid antigens presented by CD1 d molecules. On activation, NKT cells rapidly produce large amount of IFN-γ, a major cytokine of T_H_1 immune responses that inhibits HCV replication through a noncytolytic mechanism [35-37], or IL-4 and IL-13, the major cytokines of T_H_2 responses [38]. NKT cells are a link between innate and adaptive immunity exerting strong regulatory activity and producing profibrotic cytokines (IL-4 and IL-13) crucial for cirrhosis progression [38,39].

Both HCV-specific IFN-γ-producing CD8+ T cell response and a strong proliferative CD4+ T-cell response are generated during the first 6 months after infection [30,40,41]. A persistent CTL activity has been detected in patients in which HCV infection was cured but not in patients with chronic HCV infection, indicating that the CTL response has a key role in the clearance of the virus [42,43].

### Immunoregolatory cells

Much attention has recently focused on regulatory T cells (T_regs_) being able to secrete inhibitory cytokines such as IL-10 or TGF-β [44], even if their contribution is yet unclear [4]. Increased T_reg _cells were found in peripheral blood of HCV-infected patients [45-47] as well as in the tumor microenvironment of HCC patients [48]. The frequency of naturally arising CD4^+^CD25^high+ ^T_regs _in the periphery of HCV-infected patients was reported to be higher than that in patients who resolved the infection or uninfected controls [46]. T_H_1 cytokines are generally up-regulated in patients with HCC, resulting in higher levels of pro-inflammatory cytokines, as IL-1β, IL-15, IL-18, TNF-α, TNF-αRs, TNF-αRI, TNF-αRII, and IL-6 in comparison with healthy individuals [49]. However, the intra/peri-tumoral cytokines levels are often different from the serum levels [50]. Higher serum IL-6 level was an independent risk factor for HCC development in female but not male chronic hepatitis C patients [51]. IL-10 was highly expressed in HCC tumors and serum, correlating with disease progression [50]. Budhu and Wang reviewed the association between cytokine abnormalities and HCC patients and found that a dominant T_H_2-like cytokine profile (IL-4, IL-8, IL-10, and IL-5) and a decrease in the T_H_1-like cytokines (IL-1α, IL-1β, IL-2, IL-12p35, IL-12p40, IL-15, TNF-α, and IFN-γ,) was associated with the metastatic phenotype of disease [50]. Thus, it has been hypothesized that T_H_1 cytokines are involved in tumor development, whereas T_H_2 cytokines in tumor progression. Preliminary data showed that pro-inflammatory molecules (IL-1α, IL-6, IL-8, IL-12p40, GM-CSF, CCL27, CXCL1, CXCL9, CXCL10, CXCL12, β-NGF) resulted significantly up-regulated in patients affected by HCC with chronic HCV-related hepatitis and liver cirrhosis [52].

## Chronic inflammation and systemic oxidative stress

The network linking HCV infection, inflammation, free radical production, and carcinogenesis is clearly detectable in HCV-mediated chronic liver damage [53].

**Figure 2:**
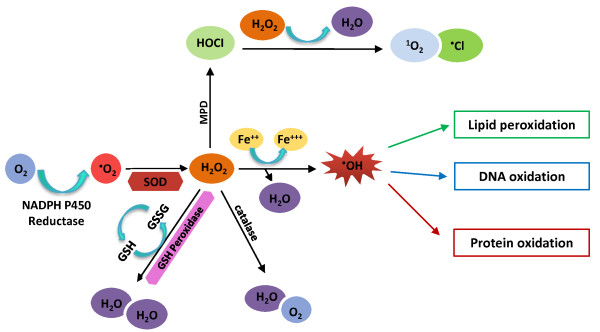
**Reactive oxygen species**. Cells generate aerobic energy by reducing molecular oxygen (O2) to water. During the metabolism of oxygen, superoxide anion (^.^O2) is formed in presence of NADPH P450 reductase. After superoxide dismutase (SOD) is added to the system, superoxide undergoes dismutation to hydrogen peroxide (H_2_O_2_), which is converted by glutathione peroxidase or catalase to water. MPD (myeloperoxidase) converts H_2_O_2 _in neutrophils to hypochlorous acid (HOCl), a strong oxidant that acts as a bactericidal agent in phagocytic cells. During a Fenton reaction, Fe^2+ ^is oxided to Fe^3+ ^and H_2_O_2 _is converted in the highly reactive hydroxyl radical ·OH. This radical is involved in lipid peroxidation, DNA and protein oxidation.

The main sources of reactive species in cells are mitochondria, cytochrome P450 and peroxisome. Under physiological conditions, there is a constant endogenous production of reactive oxygen and nitrogen species (ROS and RNS) that interact as ''signaling'' molecules for metabolism, cell cycle and intercellular transduction pathways [54]. To control the balance between production and removal of ROS, as hydroxyl and superoxide radicals, and RNS, as nitric oxide (NO), peroxynitrite and S-nitrosothiols, there are a series of protective molecules and systems globally defined as ''antioxidant defences''. Oxidative stress occurs when the generation of free radicals and active intermediates in a system exceeds the system's ability to neutralize and eliminate them. In these conditions, ROS and RNS affect the intracellular and intercellular homeostasis, leading to possible cell death and regeneration. Among ROS, the hydroxyl radical is the most damaging radical (Figure 2). It is involved in lipid peroxidation, DNA and protein oxidation and induces cell membrane damage, gene mutations, gene damage implicated in cell growth, cell-cycle, apoptosis, increase of 4-hydroxynonenal and 8-hydroxydeoxyguanosine, disruption of DNA repair pathways.

In the case of liver chronically infected by HCV [55] the virus induces reactive oxygen species (ROS) [56], and compromise the repair of damaged DNA, rendering cells more susceptible to spontaneous or mutagen-induced alterations, the underlying cause of liver cirrhosis and hepatocellular carcinoma [56]. Therefore, free radical production, oxidative genomic injury, constitutes the first step of a cascade of epigenetic (aberrant DNA methylation), genomic (point mutations) and post-genomic (protein oxidation and cytokine synthesis) events that lead to HCC [57-59]. Initially ROS interact directly with DNA, damaging specific genes that control cell growth and differentiation, cell-cycle, apoptosis, lipid peroxidation, and DNA damage repair [60]. Moreover, patients infected with HCV show increase in lipid peroxidation levels [61,62], 4-hydroxynonenal and 8-hydroxydeoxyguanosine [63-65]. Increased levels of ROS/RNS are associated with decreased antioxidant levels [63,64]. Therefore, the increased generation of reactive oxygen and nitrogen species, together with the decreased antioxidant defense, promote the development and progression of hepatic and extrahepatic complications of HCV infection [66].

Interestingly, the presence of ROS and RNS is higher in patients infected with HCV than HBV. ROS play also an important role in fibrogenesis throughout increasing platelet-derived growth factor [56] or the secretion of profibrotic cytokines, such as TGF-β. A recent proteomic study of liver biopsies from HCV infected patients at different stages of fibrosis revealed a correlation between the down-regulation of antioxidant proteins and the later stages of liver fibrosis, consistent with a role of oxidative stress in the progression of liver fibrosis and cirrhosis [67,68].

## Current HCC treatment

### Surgery

Despite surgery or liver transplant can successfully cure small or slow-growing tumors, few therapeutic options are available for advanced disease with negligible clinical benefit. For HCV-related HCC the curative therapy is surgery, either hepatic resection or liver transplantation; patients with single small HCC (< 5 cm) or up to three lesions < 3 cm should be referred for these treatment. Only 10-20% of HCC patients are candidates for surgery because of tumor size, multifocality, vascular invasion, or hepatic functional failure. In addition for patients resected, the recurrence rate can be as high as 50%[1]. Although liver transplantation has been successful for the treatment of early-stage liver cancer, a small number of HCC patients qualifies for transplantation due to donor organ shortage as well as the rapid and frequent recurrence of HCC in the transplanted liver.

### Systemic Therapy

At present, there is no effective systemic chemotherapy for HCC. Sorafenib, a vascular endothelial growth factor receptor tyrosine kinase inhibitor, has been approved by the United States Food and Drug Administration for the treatment of unresectable HCC; recent studies indicate that it is able to prolong the median survival time by nearly three months in patients with advanced HCC [1,2], but severe adverse effects, including a significant risk of bleeding, compromised these results [3].

### Alternative treatment modalities

Alternative treatment modalities including transcatheter arterial chemoembolization, targeted intra-arterial delivery of Yttrium-90 microspheres, percutaneous intratumor ethanol injection, and radiofrequency ablation are primarily for palliation and are applicable only to patients with localized liver tumors [69].

## Antioxidants role in HCC chemoprevention

In view of the limited treatment and poor prognosis of liver cancer, preventive approaches, notably surveillance and chemoprevention, have to be considered as the best strategies in lowering the current morbidity and mortality associated with HCC [15]. Given the strong association between etiologic agents, chronic liver disease (hepatitis and cirrhosis), and progression to hepatocellular carcinoma, individuals (and groups) with known risk factors must be monitored on a regular basis to detect early cancerous lesions. A number of chemopreventive agents have been examined in HCC by in vitro and in vivo studies, both in animal models and in humans.

In particular, from some studies, conducted both in vivo and in vitro, resveratrol emerged as a promising molecule that inhibits carcinogenesis with a pleiotropic mode of action [70] affecting cellular proliferation and growth, apoptosis, inflammation, invasion, angiogenesis and metastasis [71,72]. This molecule is present in grapes, berries, peanuts as well as red wine at different concentrations; in fact, red grapes provide between 0.24 and 1.25 mg of resveratrol per cup whereas boiled peanuts provide between 0.35 and 1.28 mg of resveratrol. Also red wines contain the most, at 1.92-12.59 mg per liter. Some studies report that the daily successful dosage of resveratrol is between 20 and 50 mg [70]. For this molecule there are multiple effects and action mechanism; in fact, several investigations indicated that the resveratrol has anti-HCC actions due to inhibition of abnormal cell proliferation and apoptosis through cell cycle regulation [71,72] whereas other studies reported that it can suppress the growth of HCC cells and prevent hepatocarcinogenesis by mitigating oxidative stress [70].

### In vitro studies

Since overexpression of COX-2 was demonstrated in patients with HCC, especially in nontumorous tissue with cirrhosis and well-differentiated tumorous tissue, in vitro studies have revealed that both NS-398, a selective COX-2 inhibitor, and sulindac, an analog of nonsteroidal anti-inflammatory drugs, effectively inhibit growth of human hepatoma cell lines, which is mediated by a decreased rate of cell proliferation [73]. Recent evidence suggested that cyclooxygenase-2 (COX-2)-derived prostaglandin PGE(2) and Wnt/beta-catenin signaling pathways are implicated in hepatocarcinogenesis and reported that omega-3 polyunsaturated fatty acids (PUFA), docosahexaenoic acid (DHA), and eicosapentaenoic acid (EPA) inhibited HCC growth through simultaneously inhibition of COX-2 and beta-catenin [74]. Some studies examined the possible combined effects of acyclic retinoid (ACR) plus Valproic acid (VPA) in HepG2 human HCC cell line. In particular, VPA is a histone deacetylase inhibitor (HDI), induces apoptosis and cell cycle arrest in cancer cells and enhances the sensitivity of cancer cells to retinoids. Their combination synergistically inhibited the growth of HepG2 cells without affecting the growth of normal human hepatocytes and increased the expression of RARβ and p21(CIP1), while inhibiting the phosphorylation of RXRα. This combination resulted an effective regimen for the chemoprevention and chemotherapy of HCC [75]. Finally, the combination of 9-cis-retinoic acid (9cRA) plus trastuzumab resulted to inhibit the activation of HER2 and its downstream signaling pathways, subsequently inhibiting the phosphorylation of RXR alpha and the growth of HCC cells [76].

### In animal models

Chemopreventive agents in preclinical development stage include S-adenosyl-L-methionine [77], curcumin [78], a 5a-reductase inhibitor [79], vitamin E [80], vitamin D [81], and green tea [82], as well as a number of herbal extracts. Moreover, the preventive effect of flavonoids, quercetin or Acacia nilotica bark extract (ANBE) via oxidant/antioxidant activity was demonstrated on hepatic cancer in rats [83-85]. Recently several other molecules with antioxidative properties were evaluated (for example, Siraitia grosvenorii extract, black tea polyphenols, xanthohumol from hops (Humulus lupulus L.)) [86-88]. Also, butyric acid (BA) being a member of histone deacetylase inhibitors (HDAI) has been proposed as chemiopreventive agent. In fact some studies have tested the efficacy of tributyrin (TB), a proposed BA prodrug, on rats treated with the compound during initial phases of "resistant hepatocyte" model of hepatocarcinogenesis. TB increased hepatic nuclear histone H3K9 hyperacetylation specifically in PNL and p21 protein expression, which could be associated with HDI effects [89]. In 2008 the antiproliferative effect of gallic acid was investigated during diethylnitrosamine (DEN)-inducedHCC) in rats. Gallic acid treatment significantly attenuated some alterations (i.e. increased levels of aspartate transaminase, alanine transaminase, alkaline phosphatase, acid phosphatase, lactate dehydrogenase, gamma-glutamyltransferase, 5'-nucleotidase, bilirubin, alpha-fetoprotein, carcinoembryonic antigen) and decreased the levels of argyophillic nucleolar organizing regions (AgNORs) and proliferating cell nuclear antigen (PCNA) [90].

Several studies have investigated the effect of selenium on different phases of hepatocarcinogenesis using varying in vivo hepatocarcinogenesis protocols. Selenium is an essential mineral for both human and animals and functions as a component of several proteins, termed selenoproteins (i.e. glutathione peroxidases, thioredoxin reductates, selenoprotein P etc) [91]. The level of selenium added to the American Institute of Nutrition 93 (AIN-93) diet was 0.15 mg Se/kg diet, with the total amount estimated to be about 0.18 mg/kg diet, due to background levels in the other ingredients of the diet [92]. Several early studies observed that selenium inhibited complete carcinogenesis in the liver. It was also demonstrated that using a Solt-Farber protocol, 1 and 5 mg/kg selenium administered to rats during the initiation had no effect on the number and volume of hepatic nodules, but selenium administered during either the promotion or 6 month progression stages decreased the volume occupied by the nodules in the liver [93].

Finally, a study in 2010 on lanreotide, a somatostatin analogue, showed that it inhibits the development of "foci of altered hepatocytes", which represent very early neoplastic changes in rat liver, and decreases hepatocyte proliferation and inhibition of fibrosis in rats model [94].

### In human

In the setting of secondary chemoprevention, literature data pooling suggests a slight preventive effect of interferon (IFN) on HCC development in patients with HCV-related cirrhosis. The magnitude of this effect is low, and the observed benefit might be due to spurious associations. The preventive effect is limited to sustained virological responders to IFN [95]. In fact, α-interferon therapy leads to complete viral eradication in some long-term responders; its persistence thus depends on HCV RNA replication [96]. However, IFN reduced the risk of HCC in HCV-related liver cirrhosis [97] whereas the HALT-C study showed that long-term therapy with IFN did not reduce the rate of disease progression in patients with chronic hepatitis C and advanced fibrosis, with or without cirrhosis [98]. Overall, the best long-term benefit of IFN is seen almost exclusively in long-term virologic responders, since no significant differences between treated patients and untreated patients, [99]. Annual incidence of HCC in HCV-related cirrhotic or pre-cirrhotic liver is reported as 4-8%, and IFN-α treatment is estimated to reduce approximately 50% of annual incidence of HCC in chronic hepatitis C with cirrhotic or pre-cirrhotic liver, if SVR rate of approximately 30% is achieved. Preventive effect of IFN-alpha on HCC development is considered because of anti-necroinflammatory effect and suppression of viral replication. Furthermore, SVR leads to the regression of histological fibrosis, even in cirrhotic liver [100].

Glycyrrhizin, an aqueous extract of licorice root, was reported to decrease the risk of HCC in HCV-infected individuals [101] as well as medicinal ginseng was tested for HCC-preventive capability among HCV-infected Japanese patients [102]. A study on vitamin A (retinol) showed that low levels of retinol were present up to five years before HCC diagnosis among individuals who developed this disease [103].

Muto *et al *randomly assigned 89 HCC patients who were cancer free following resection or ablation to receive polyprenoic acid, an acyclic retinoid, and showed that the recurrence rate was about 50% lower in the retinoid treated group [104,105].

The role of selenium was investigated also in chemoprevention. Several studies have investigated on HCV-associated HCC patients the selenium (Se) effect, In particular, most of selenium supplementation trials were based in China and the remaining trials were in the USA, Italy and India. The first China trial found that selenium supplementation using table salt fortified with sodium selenite (30-50 mg Se/day) resulted in an almost 50% decrease in the primary liver cancer incidence [106]. Another study showed that selenite-fortified salt supplementation reduced the incidence rate of viral infectious hepatitis [107]. Yu et al [106] reported also a significant decrease in primary liver cancer among those receiving selenium yeast compared with controls.

However other epidemiological studies have demonstrated that higher serum level of other antioxidants do not seem to correlate with liver cancer prevention. In fact, in a population-based 11.7-year follow-up study on mortality rates from cancer in a Japanese population, higher serum tocopherol (vitamin E) levels did not correlate with reduced risk of mortality from liver cancer [108]. Moreover, in a 15-year follow-up prospective study in males, high serum levels of tocopherols did not reduce the risk of developing HCC [109]. One epidemiological study has examined the role of dietary vitamin C in liver cancer etiology. In that prospective study, Kurahashi et al [110] examined the effect of the consumption of fruit, vegetables, and some antioxidants on the risk of HCC. Intake of vitamin C in the middle and highest tertile were found to significantly increase the risk of developing HCC in smokers, whereas its effect in non-smokers was not significant.

## Conclusions

HCC is unique among cancers occurring mostly in patients with chronic inflammation and cirrhosis. Its treatment is challenging since HCC is largely refractory to chemotherapy and are often silent until local tumor spread or distant metastasis. Thus, HCC prevention might represent the best opportunity to reduce the worldwide burden of disease. Although HBV vaccination will reduce the number of individuals at risk for HCC development, a tremendous number of people are currently at elevated risk for HCC due to HCV-correlated chronic hepatitis and/or cirrhosis. This population with known risk factors has to be monitored on a regular basis to detect early cancerous lesions (surveillance and eventual treatment). Detection and diagnosis of HCC at an early stage may significantly improve the survival of patients with this disease. Hence, there is also an obvious critical need to develop alternative strategies to prevent HCC development. In fact the HCC chemoprevention may be aimed to develop new preventive strategies for reducing inflammation rather than virus replication. Unfortunately there are limited epidemiological data linking increased levels of several antioxidants with HCC prevention. In fact, human studies do not provide compelling evidence that consuming higher amounts of some studied antioxidants would decrease one's probability of developing HCC. This suggests that further studies are needed to develop clinically effective chemopreventive agents impairing chronic inflammatory process underlying cancer. Moreover further insight into the mechanism of chemopreventive agents drugs will likely to unveil that microenvironment (vasculature, chemokine, immuneregulatory cells) is among targets of chemopreventive agents.

## List of abbreviations

CLDN1: claudin; CTL: cytotoxic T lymphocytes; DC: Dendritic Cells; HBV: Hepatitis B Virus; HCC: Hepatocellular Carcinoma; HCV: Hepatitis C Virus; HDL: High-Density Lipoprotein; iDC: immature Dendritic Cells; IFN: interferon; IL: interleukin; ISGs: IFN-stimulated genes; LDL: Low-Density Lipoprotein; mDCs: Mature Dendritic Cells; MHC: Major Histocompatibility Complex; NF-κ;B: nuclear factor κ;B; NK: natural killer cells; NKT: natural killer T cells; PAMP: pathogen-associated molecular pattern; SR-BI: scavenger receptor class B type I; TCR: T cell receptor; TGF: transforming growth factor; T_H_: T helper cells; T_H_0: naive T cells; T_H_1: T helper type 1; T_H_2: T helper type 2; TNF: tumor necrosis factor; TLR: Toll-like receptors; VLDL: Very Low Density Lipoprotein

## Competing interests

The authors declare that they have no competing interests.

## Authors' contributions

SS and CG have contributed to conception and design of the review.

SS, CS and CG are involved in drafting the manuscript and have given final approval of the version to be published.
